# Internal jugular venous thrombosis due to Trousseau’s syndrome as the presenting feature of metastatic prostate carcinoma: a case report

**DOI:** 10.1186/s13256-016-0884-9

**Published:** 2016-04-21

**Authors:** Asela Rasika Bandara, Harith Wimalarathna, Ranjith Kalupahana, Sonali Sihindi Chapa Gunathilake

**Affiliations:** Teaching Hospital, Kandy, 20000 Sri Lanka

**Keywords:** Internal jugular vein thrombosis, Migratory thrombophebitis, Trousseau’s syndrome, Hypercoagulability, Prostate cancer

## Abstract

**Background:**

Internal jugular vein thrombosis is a rare vascular event with a potentially fatal outcome. Of the known etiologies, internal malignancies, either known or occult, are well described. Even though malignancies are known to present with internal jugular vein thrombosis, it rarely occurs due to prostate carcinoma. Many cases of jugular vein and superior vena cava thrombosis secondary to malignancies are due to metastatic compression of veins. Recurrent and unusual vascular thrombosis due to hypercoagulability associated with malignancies is also known as Trousseau’s syndrome. Here we report a rare case of a patient with internal jugular vein thrombosis as a presenting feature of metastatic prostate carcinoma, which is a case of Trousseau’s syndrome.

**Case presentation:**

A 75-year-old Sri Lankan man with hypertension, hyperlipidemia, and past history of spontaneous intracranial hemorrhage presented with a short history of painless swelling in his left supraclavicular fossa. An examination revealed the swelling was due to a thickened left external jugular vein. A duplex ultrasound scan revealed left-sided internal jugular, external jugular, and brachiocephalic venous thrombosis. Surveillance into underlying malignancies showed an irregular, hard prostate gland suspicious of prostate carcinoma, which was proven with histology, and biochemically. A computed tomography scan found extensive vertebral, pelvic bone, intra-abdominal lymph node metastasis, and a single right-sided lower lung metastatic lesion, with no direct involvement of the jugular vein.

**Conclusions:**

Spontaneous thrombosis of the internal jugular vein due to Trousseau’s syndrome is rare and unusual. Clinicians should promptly investigate for malignancies as it can be the first presentation of underlying occult malignancies. Although prostate carcinomas are rare to present with internal jugular vein thrombosis, this case illustrates the importance of having a high degree of suspicion in the appropriate clinical setting.

## Background

Internal jugular venous thrombosis is a rare vascular event that occurs secondary to prolonged central venous catheterization, intravenous drug abuse, head and neck infections, trauma, and hypercoagulable status [1–3]. Rarely, spontaneous internal jugular vein thrombosis can be the presenting feature of underlying malignancy, known or occult [4]. Suggested mechanisms are (1) direct effect by the metastatic tumor, (2) as a result of hypercoagulable status and migratory thrombophebitis, generated secondary to the underlying malignancy, known as Trousseau’s syndrome. Even though internal jugular venous and superior vena cava thromboses have been reported in association with prostate carcinoma, many of them are due to the mechanical effects of metastatic tumors. However, we report the case of a patient with spontaneous internal jugular venous thrombosis extending to the external jugular and brachiocephalic veins, as a result of Trousseau’s syndrome, as a presenting feature of metastatic prostate carcinoma.

## Case presentation

A 75-year-old Sri Lankan man with past history of hypertension and hyperlipidemia of 6-year duration and spontaneous right parietal intracranial hemorrhage 6 years ago with near normal recovery presented with painless swelling in his left supraclavicular area for the past 10 days. There had been no trauma or interventions done involving his neck and upper limb in the recent past. He did not have a history suggestive of ear, nose, throat, or oral infections and denied dyspnea, hemoptysis, back pain, lower urinary tract symptoms, or constitutional symptoms.

An examination revealed a nontender swelling in his left supraclavicular fossa due to a cord-like thickening of the left external jugular vein. There were no signs of infection either locally or involving his left upper limb. An examination of his oral cavity, ear, nose, and throat was unremarkable, and there was no lymphadenopathy. His upper limbs and face did not show evidence of venous congestion. Examinations of his cardiovascular, respiratory, central nervous, musculoskeletal and abdominal system were normal, but a digital examination of his rectum revealed a moderately enlarged, hard, irregular prostate gland.

**Fig. 1:**
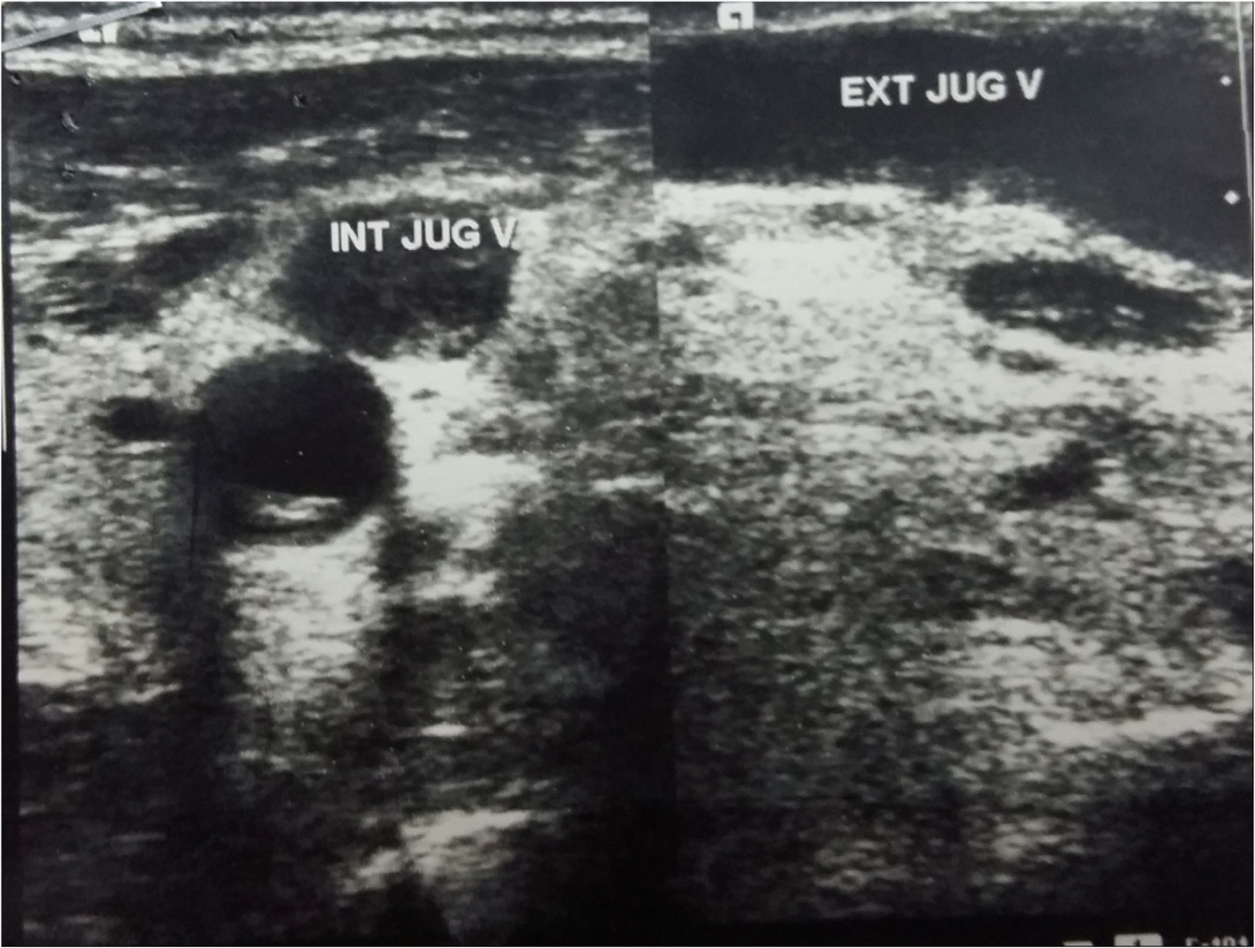
Ultrasound duplex scan of neck showing thrombosis of internal jugular vein. *EXT JUG V* external jugular vein, *INT JUG V* internal jugular vein

A venous duplex ultrasound scan of his neck confirmed thrombosis of his left internal jugular, extending into his external jugular and brachiocephalic veins (Fig. 1). A contrast-enhanced computed tomography (CT) scan of his neck and upper chest demonstrated patent superior vena cava and right jugular veins, but his left internal jugular, external jugular, and left brachiocephalic veins were not visualized. However, it was unable to show thrombosis of these nonvisualized veins, as the study was done in the arterial phase. A chest radiograph did not show any masses or mediastinal widening.

His hematological investigations showed a hemoglobin of 11.4 g/dL, a white cell count of 8170/ mm3 (neutrophil 66 %, lymphocyte 21 %, middle fraction 13 %) and a platelet count of 530,000/mm3. The blood picture revealed evidence of inflammation. His erythrocyte sedimentation rate was 40 mm in the first hour (normal <20). His C-reactive protein level was 3 mg/l (normal <10 mg/L). A blood culture revealed no growth at 48 hours. Coagulation studies with activated partial thromboplastin time (APTT) and prothrombin time (PT) were normal. Thrombophilia screening was normal (antinuclear antibodies, anticardiolipin antibodies were not detected). His renal functions were normal, but liver tests revealed alkaline phosphatase (ALP) of 215 IU/L (53–128 IU/L) and gamma-glutamyltransferase (GGT) of 222 IU/L (normal <31 IU/L), while other enzymes were within normal range. His serum calcium was significantly elevated to 3.9 mmol/l. Human immunodeficiency virus (HIV) antibodies and venereal disease research laboratory (VDRL) test results were negative.

**Fig. 2:**
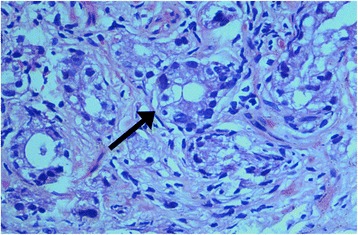
Histology of prostate adenocarcinoma showing an abnormal gland with a irregular, thick wall, made up of cells with abnormally large nuclei (arrow)

His prostate-specific antigen was greatly elevated to 257 ng/ml (normal <4 ng/ml). An ultrasound scan of his pelvis revealed a heterogeneously enlarged prostate with central calcification. A prostate biopsy revealed 30 % of tissue infiltrated by a conventional prostate adenocarcinoma of acinar type, which is predominantly composed of small glandular structures arranged in ill-defined nodules, giving the primary Gleason morphologic grade of 3, and less commonly of cribriform structures and large irregular glands, giving the secondary pattern as Gleason morphology grade 4. Perineural invasion, lymphovascular emboli or extraprostatic tissue infiltration was not demonstrated in the sections examined (Fig. 2). There was a single lung metastasis in the lower lobe of his right lung, but no pleural effusion. There was no evidence of cervical or mediastinal metastasis.

He was immediately started on anticoagulation therapy with intravenous heparin followed by oral warfarin, to maintain his international normalized ratio (INR) 2–3. He showed a good response with treatment resulting in symptomatic relief and resolved neck swelling. He did not develop further thrombotic episodes while on anticoagulation therapy. He is currently being treated at the oncology unit with a chemotherapeutic regimen, which includes docetaxel and bicalutamide.

## Discussion

Internal jugular venous thrombosis was first described by Long as a complication of peritonsillar abscess in 1912 [5]. It is a rare, but potentially fatal vascular event [1]. Central venous catheterization is the commonest etiology in modern clinical practice [4]. A review done by Shakeel *et al*. illustrated many other causes including deep head and neck infections, intravenous drug abuse, hypercoagulable disorders, ovarian hyperstimulation, and malignancies [6].

Central venous thrombosis in association with malignancies may occur as result of (1) local disturbance to venous flow due to malignancy itself or metastatic deposits, or (2) due to hypercoagulability associated with malignancies. Migratory thrombophebititis due to distant malignancies, known as Trousseau’s syndrome can cause thrombosis in unusual sites including internal jugular veins and superior vena cava [7]. Several factors are responsible for hypercoagulability in malignancies. These include (1) increased procoagulant factors (for example, tissue factor, cancer procoagulant), (2) reduced anticoagulant factors (for example, antithrombin, protein C, protein S, tissue factor pathway inhibitor), (3) altered fibrinolysis through elevated plasminogen activator inhibitor, (4) expression of tissue factor and cytokines, which activate coagulation cascade, (for example, tumor necrosis factor, interleukin 1 and 6) [8]. Sack *et al*. reviewed 541 patients with Trousseau’s syndrome and reported that underlying malignancy was found in lung (25.6 %), pancreas (17.4 %), stomach (16.8 %), colon (15.2 %), prostate (6.5 %), and others [9].

Prostate carcinoma has been shown to cause internal jugular venous or superior vena cava thrombosis in few case reports. [7, 10–13]. Except for the case report published by Takeda *et al.* [7] where superior vena cava thrombosis had occurred as a nonmetastatic manifestation, all the other cases had venous thrombosis in association with mediastinal metastasis of prostate carcinoma. As there was no cervical or mediastinal metastasis in our patient, internal jugular thrombosis in this case is likely to be due to hypercoagulability and Trousseau’s syndrome secondary to distant prostate carcinoma. Because large central veins are valveless and they have an elastic wall, allowing alternative collapse and expansion by the action of respiration and heart pumping, Trousseau’s syndrome in head and neck veins is rare [14], making our case an unusual presentation of prostate carcinoma.

Contrast venography is the gold standard investigation for diagnosis of internal jugular vein thrombosis. But duplex ultrasound provides a more cost-effective, portable, easily available diagnostic method with excellent accuracy, without radiation [4]. However, ultrasonography lacks the ability to provide detailed information regarding the surrounding tissue and often needs to be followed by CT and magnetic resonance imaging (MRI), which provide valuable information for identifying the underlying pathology [6].

Treatment of internal jugular venous thrombosis is anticoagulation therapy, initially with intravenous heparin followed by long-term anticoagulation therapy with oral warfarin. However, the definitive treatment should involve treatment of the underlying malignancy [2].

## Conclusions

Spontaneous thrombosis of the internal jugular vein is extremely rare and an unusual presentation. Prompt diagnosis and treatment is necessary to prevent serious complications. Patients should be investigated for malignancies as it can be the first presentation of underlying distant malignancies. Although prostate carcinomas are rare to present as internal jugular vein thrombosis due to Trousseau’s syndrome, this case emphasizes the importance of awareness among physicians regarding rare presentations of internal malignancies for correct diagnosis and appropriate management.

## Consent

Written informed consent was obtained from the patient for publication of this case report and any accompanying images. A copy of the written consent is available for review by the Editor-in-Chief of this journal.

## Abbreviations

ALP: alkaline phosphatase
APTT: activated partial thromboplastin time
CT: computed tomography
GGT: gamma-glutamyltransferase
HIV: human immunodeficiency virus
INR: international normalized ratio
MRI: magnetic resonant imaging
PT: prothrombin time
VDRL: venereal disease research laboratory
